# Primary Hepatic Lymphoma Presenting as an Acute Abdomen in a Young Female Patient: A Case Report and Literature Review

**DOI:** 10.1155/2019/6784325

**Published:** 2019-07-30

**Authors:** Walid E. Abdelrahim, Kamal E. Mohamed, Salwa O. Mekki, Eltaib A. Saad

**Affiliations:** ^1^Department of Surgery, Soba University Hospital, University of Khartoum, Sudan; ^2^Department of Clinical Oncology, Soba University Hospital, University of Khartoum, Sudan; ^3^Department of Pathology, Soba University Hospital, University of Khartoum, Sudan; ^4^Department of Surgery, Midland Regional Hospital Mullingar, Ireland

## Abstract

We report a rare case of primary hepatic lymphoma (PHL) in a hepatitis B virus- (HBV-) infected young female patient who presented with right upper abdominal pain, nausea, and vomiting for a few days. The preoperative diagnosis was difficult due to the rarity of the disease and the presence of a solitary hypodense mass in the left lobe of the liver on contrast-enhanced computed tomography (CT) scan with a normal alpha-fetoprotein (AFP) and negative cytology. She underwent an uneventful extended left hemihepatectomy, and the surgical biopsy revealed a PHL—of diffuse large B-cell lymphoma (DLBCL) type—with negative resection margins. She received adjuvant combination chemotherapy and remained disease-free with normal serial radiology over a 2-year follow-up period.

## 1. Introduction

Primary hepatic lymphoma (PHL) is an unusual lymphoma which is confined to the liver with no evidence of extrahepatic lymphatic involvement in the spleen, lymph nodes, bone marrow, or other lymphoid structures [1, 2]. It is a rare malignancy accounting for less than 0.4% of all extranodal non-Hodgkin lymphoma (NHL) and represents about 0.016% of all NHL [1]. The vast majority of PHL are NHL, most often a diffuse large B-cell lymphoma (DLBCL) type [1, 2]. PHL has a fairly nonspecific pattern of clinical presentation and laboratory and radiological findings [1–3]. The exact etiology of PHL is largely unknown, although associations have been linked to viral infections, immunosuppression, liver cirrhosis, primary biliary cirrhosis, and autoimmune diseases [3].

Owing to its rarity, there is a lack of established guidelines on the optimal management of PHL [4, 5]. The prognosis has been generally described as poor, but favorable outcomes were reported recently with employing combination chemotherapy regimens and surgical resection for operable tumors [6–10].

We present a case of PHL in an HBV-infected young female patient who presented with right upper abdominal pain and vomiting for a few days. A computed tomography (CT) scan revealed a solitary hypodense solid mass confined to the left lobe of the liver. Preoperative diagnosis was difficult given the findings of a normal alpha-fetoprotein (AFP) level and negative ascites aspirate cytology. She underwent an extended left hemihepatectomy. The histopathology revealed a primary hepatic DLBCL with negative resection margins. She received an adjuvant combination chemotherapy regime and remained disease-free over a 2-year follow-up period.

## 2. Case Presentation

A 19-year- old South Sudanese female patient presented to our emergency department with right upper abdominal pain and vomiting for a few days prior to presentation. She denied symptoms of fever or rigors, sweating, jaundice, hematemesis, epistaxis, itching, anorexia, or loss of weight. She had no similar presentations with such symptoms. The rest of the systemic review was otherwise unremarkable. She reported being a nonsmoker and nondrinker. The past medical history and family history were insignificant. Physical examination revealed a distressed and pale young patient who was not jaundiced or febrile with stable vital signs. Cervical lymph nodes were not palpable. Abdominal examination revealed a mildly distended abdomen with tender hepatomegaly. Murphy's sign was negative.

General investigations showed a low Hb of 9 g/dl with a normal total and differential WCC count and platelet count. Serum electrolytes, renal profile, liver function parameters (i.e., liver enzymes (AST, ALT, and ALP); total bilirubin; and albumin), and coagulation profile were all within the normal ranges. HBV serology was positive and was confirmed by polymerase chain reaction (PCR). HCV and HIV screening serology was negative. The iron profile was normal.

**Figure 1:**
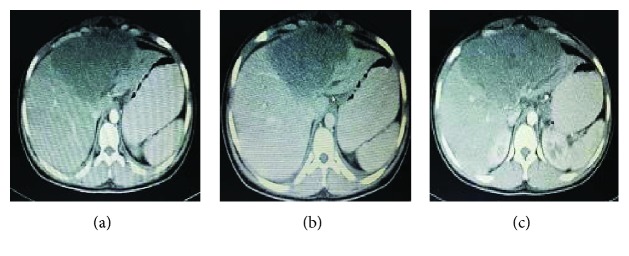
Contrast-enhanced CT abdomen. (a) Early arterial phase showing a hypodense lesion in the left liver lobe and a normal spleen. (b) Venous phase showing a hypodense lesion confined to the left liver lobe and firmly attached to the middle hepatic vein. (c) A mixed-enhanced pattern of the lesion on the delayed phase.

Abdominal US scan demonstrated a 10X13 cm hypoechoic mass arising from the left lobe of the liver and mild ascites. An US-guided cytology aspiration of the ascites was negative for any malignant cells. A triple-phase contrast-enhanced CT abdomen depicted a 14 × 14 cm well-defined left liver lobe mass, involving segments II, III, and VI-A and VI-B, which was hypodense in the early arterial phase, retaining the contrast in the venous phase, and showing a mixed-enhanced pattern in the delayed phase (Figures 1(a)–1(c)). The spleen was remarkably normal, and the mesenteric, para-aortic, and retroperitoneal lymph nodes were not enlarged. The radiological appearances suggested a primary hepatic tumor; however, the AFP level was within the normal range.

The possibility of a metastatic liver tumor was less likely given the acute onset of the symptoms, the lack of clinical features that would suggest a primary breast, pulmonary, or gastrointestinal origin, and the radiological appearance of the mass which was not keeping with a metastatic lesion and, similarly the biochemical findings, as the carcinoembryonic antigen (CEA) level was within the normal limits. Nevertheless, a further work-up was carried out to outrule a possible primary origin. An upper endoscopy was performed as the patient presented with an upper abdominal pain, and it was normal. CT scans of the chest and neck were negative for primary pulmonary lesions, and the hilar, mediastinal, and cervical lymph nodes were not enlarged. A bone marrow biopsy revealed a normal cellularity pattern.

**Figure 2:**
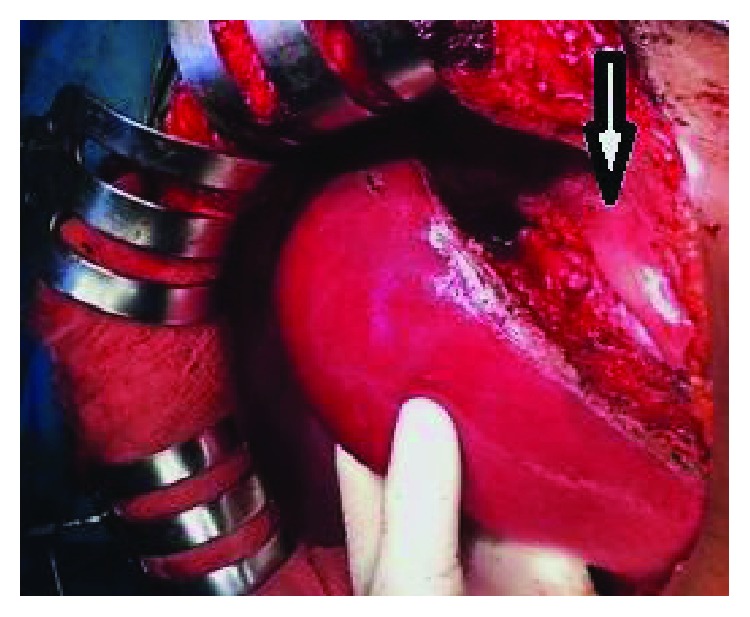
An operative image shows a normal right liver lobe, and a well-demarcated mass arising from the left lobe was resected (vertical arrow).

The patient's clinical condition deteriorated, and she experienced worsening abdominal signs on day 6 of admission. A diagnosis of peritonitis secondary to ruptured liver mass was suggested, and a decision of surgical exploration was made after a multidisciplinary team (MDT) discussion. Intraoperatively, a mild ascites was noted but the peritoneum was grossly free of any deposits or seedlings. A well-demarcated mass was palpated in the left lobe of the liver as characterized on CT scan (Figure 2). Notably, no enlarged lymph nodes were encountered during the dissection of the porta hepatis or hepatoduodenal ligaments. The patient underwent an extended left hemihepatectomy with an uneventful postoperative course. Macroscopic examination of the surgical biopsy revealed a 15 × 15 cm solid mass with well-demarcated margins.

**Figure 3:**
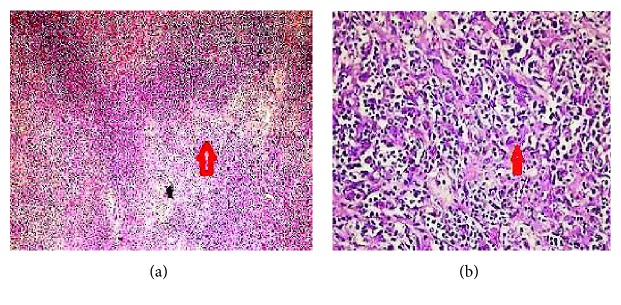
(a) Hepatic infiltration with lymphoid cells (a population of dark blue-rounded cells—vertical red arrow) (H&E-stained slide (×20)). (b) Uniform sheet of round B-lymphoid cells (vertical red arrow) (H&E-stained slide (×100)).

Histopathology revealed a hepatic infiltration with lymphoid B-cells consistent with primary hepatic diffuse large B-cell lymphoma (DLBCL) with negative resection margins (Figures 3(a) and 3(b)). Immunohistochemistry was positive for CD20 and CD45 and negative for CD2, CD3, CD15, and CD30. Prognostic markers (lactate dehydrogenase (LDH), serum calcium, and beta-2 microglobulin) were all within the normal limits.

She was referred to the oncology team for adjuvant chemotherapy. She received six cycles of R-CHOP regime (Adriamycin, bleomycin, vinblastine, and dacarbazine) delivered every three weeks. She tolerated that regime fairly well except mild nausea and vomiting. However, upon completion of the course, she was readmitted with an acute liver failure due to HBV flare-up, presumably due to immunosuppression, and she was kept in the ICU for 10 days for supportive management and antiviral therapy; she made a slow recovery over 4 weeks, and the liver functions returned to normal without permanent damage. She was placed on a long-term antiviral therapy (entecavir) per hepatology team's advice. She remained disease-free with normal serial radiology over a 2-year follow-up period.

## 3. Discussion

PHL is a rare disease entity despite the fact that the liver is being a relatively common site for extranodal involvement in patients with systemic NHL [1, 2], as the secondary liver involvement is detectable in more than half of the patients with systemic NHL [2]. Kit Lei [11] defined three diagnostic criteria for PHL: (I) at the time of disease presentation, the patient's symptoms are caused by the liver involvement; (II) there is an absence of palpable lymphadenopathy, and there is no radiologic evidence of distant lymphadenopathy; (III) there is an absence of leukemic blood involvement in the peripheral blood smear [11]. These specific criteria would explain the importance of a careful diagnostic work-up to exclude any systemic lymphatic involvement among those suspected of having PHL [4].

The etiology of PHL remains largely unknown [4, 5]. A number of possible etiologic factors have been proposed in the literature, i.e., viral infections, for instance, HBV, HCV, HIV, and Epstein–Barr virus (EPV), liver cirrhosis, primary biliary cirrhosis, immunosuppressive therapy, and autoimmune disease [1, 2, 4, 5]. Few reports, however, described an occurrence of PHL without the abovementioned presumed risk factors [4]. Our patient was HBV-positive, and this viral infection would be the probable risk factor for PHL. The literature described PHL's occurrence mainly among middle-aged groups with a male to female ratio of 2.3 : 1 [1, 2]. The younger age of onset in our young patient would be possibly explained by the early age of infection with hepatitis B. Nevertheless, conflicting data exist on the presumed association between HBV infection and PHL occurrence [1]. At present, it is probable that any suggested association between PHL and HBV is likely coincidental rather than being causal [1].

The clinical presentation with constitutional symptoms as in this case is in keeping with the literature [1–6]. The typical B symptoms of fever and weight loss occur in about one-third of the PHL patients [1]. Being a localized disease, PHL is less frequently associated with these symptoms as compared to systemic NHL [2]. The physical findings of gross hepatomegaly with the absence of jaundice also agreed with the literature, as a clinical finding of jaundice was reported in 10-20% of cases [1].

The laboratory findings in this case with normal LFTs disagreed with many series that reported a variable pattern of mild to moderate LFT derangement [1–2]. The liver function abnormalities in PHL could possibly reflect a cholestatic or a cytolytic pathological process produced by the tumor effect [1]. Furthermore, a presentation with an acute liver failure due to a PHL was reported before in the literature [1]. Similarly, the LDH level was normal in our case, which is not in line of the literature that reported an elevated LDH level in 30-80% of cases [1]. In fact, PHL patients generally have lower LDH levels than those with secondary liver involvement in systemic NHL [2]. This finding would explain in part the assumption that the patients with PHL usually have more favorable prognosis and longer survival compared to those with systemic NHL disease [2]. A normal level of AFP is typical to PHL [2], a finding that would help in differentiating it from primary hepatocellular carcinoma and metastatic liver disease [2].

A solitary hypodense liver lesion on contrast-enhanced CT scan is reported in more than half of patients (60%) [1]. Other less common radiological findings are multiple lesions (35-40%) and a diffuse infiltration pattern (5%) which is associated with a worse prognosis [1]. PHL may be confused with primary hepatic carcinoma, metastatic liver diseases, and systemic NHL with secondary hepatic involvement due to its rarity and the noncharacteristic clinical, laboratory, and radiologic features [2, 6]. Imaging modalities, especially contrast-enhanced CT scan and gadolinium-enhanced MRI scan, aid in further characterization of the liver lesions [1]. Nevertheless, the pathological diagnosis remains the gold standard to reach the final diagnosis [1]. DLBCL is the most common pathological variant [1–6]; other reported variants include high-grade lymphoma (17%), follicular lymphoma (4%), diffuse histiocytic lymphoma (5%), anaplastic large-cell lymphoma, and T-cell-rich B-cell lymphoma [4, 5]. Immunohistochemistry and flow cytometry studies help in further characterization of the pathological subtypes [1].

PHL has been historically described as an aggressive disease with a relatively poor prognosis [11]. However, Page et al. [12] described a favorable outcome with the use of combination chemotherapy and surgery (for resectable tumors), with a reported remission rate of 83.3% [12]. Recent series reported favorable short- and midterm outcomes and longer survival rates with the use of combination chemotherapy regimens and surgical resection as well [6–9]. R-CHOP regime is associated with a prolonged survival and minimal toxicity and has thus been widely employed as an adjuvant chemotherapy regime [4]. Other authors reported a successful response to neoadjuvant chemotherapy that facilitated a subsequent surgical resection [5, 8]. Nevertheless, the current data regarding the management and prognosis of PHL is derived primarily from case reports and small series and not based on systematic reviews or randomized trials [1, 2, 11, 12].

## 4. Summary

PHL is a fairly rare disease. We presented a case of DLBCL in an HBV-infected young female patient who presented with an acute abdomen. The preoperative diagnosis was difficult due to the rarity of the disease, younger age of presentation, and presence of a solitary liver lesion on CT scan with a normal AFP level and negative cytology. Our patient underwent a surgical resection followed by combination chemotherapy and remained disease-free over a 2-year follow-up period. It is important to recognize PHL as it generally has a better prognosis than primary hepatocellular carcinoma and systemic lymphoma with secondary liver involvement.
